# The Genomic and Phenotypic Characterization of the *Sym2^A^* Introgression Line A33.18 of Pea (*Pisum sativum* L.) with the Increased Specificity of Root Nodule Symbiosis

**DOI:** 10.3390/plants14030427

**Published:** 2025-02-01

**Authors:** Anton S. Sulima, Igor Yu. Zhuravlev, Elizaveta A. Alexeeva, Marina S. Kliukova, Evgeny A. Zorin, Valeria A. Rakova, Michail L. Gordon, Olga A. Kulaeva, Daria A. Romanyuk, Gulnar A. Akhtemova, Aleksandr I. Zhernakov, Elena V. Semenova, Margarita A. Vishnyakova, Igor A. Tikhonovich, Vladimir A. Zhukov

**Affiliations:** 1 All-Russia Research Institute for Agricultural Microbiology (ARRIAM), Pushkin, 196608 St. Petersburg, Russia; asulima@arriam.ru (A.S.S.); marina.kliukova@gmail.com (M.S.K.); ezorin@arriam.ru (E.A.Z.); v.rakova@arriam.ru (V.A.R.); okulaeva@arriam.ru (O.A.K.); d.romanyuk@arriam.ru (D.A.R.); azhernakov@arriam.ru (A.I.Z.); i.tikhonovich@arriam.ru (I.A.T.); 2 Plant Biology and Biotechnology Department, Sirius University of Science and Technology, 354340 Sirius, Russia; zhuravlev.iy@talantiuspeh.ru (I.Y.Z.); lizasoma.a.a@gmail.com (E.A.A.); 3 N. I. Vavilov All-Russian Institute of Plant Genetic Resources (VIR), 190000 St. Petersburg, Russia; m.gordon.zelenoborsky@gmail.com (M.L.G.); e.semenova@vir.nw.ru (E.V.S.); m.vishnyakova@vir.nw.ru (M.A.V.)

**Keywords:** plant–microbe symbiosis, legumes, *Pisum sativum*, *Sym2*, specificity of symbiosis, introgression line, molecular breeding

## Abstract

In pea (*Pisum sativum* L.), alleles of the *Sym2* gene determine the specificity of the interaction with nodule bacteria (rhizobia). The *Sym2^A^* allele present in landraces from Afghanistan provides higher selectiveness toward rhizobia than the *Sym2^E^* allele present in European cultivars. Rhizobial strains possessing the *nodX* gene can interact with both *Sym2^A^* and *Sym2^E^* peas, while strains lacking *nodX* can interact only with *Sym2^E^* peas. Here, we studied the previously obtained introgression line A33.18 bearing *Sym2^A^* in a homozygous state in the genome of the European pea cultivar ‘Rondo’. A33.18 has proved its high selectiveness in pot experiments. Genome sequencing has shown that A33.18 possesses an 18.2 Mb region inherited from Afghanistan pea with 63 genes, including 5 receptor kinase genes, among which was the *Sym2* candidate gene *LykX*. In a field experiment, under inoculation with the *nodX^+^* strain TOM, over 95% of nodules of A33.18 contained TOM, as opposed to less than 8% of nodules containing TOM in the parental European cultivar ‘Rondo’. Thus, introgression of *Sym2^A^* enabled peas to interact specifically with the *nodX^+^* strain, favoring the formation of nodules by the strain from the inoculum and protecting peas from the indigenous soil microbiota.

## 1. Introduction

Pea (*Pisum sativum* L.) is one of the most important crops and the second most cultivated legume in the world [1,2]. It is an important source of nutrients for humans and animals, as well as an integral component of crop rotation, capable of improving soil conditions through symbiotic interactions with beneficial soil microbiota [3,4]. The most well-known and important symbiosis of pea is that with nitrogen-fixing bacteria (rhizobia), which provide the plant with access to atmospheric nitrogen, thereby allowing it to avoid the competition for sources of bound nitrogen in the soil. The establishment of this symbiosis involves mutual recognition of partners through the exchange of signaling molecules, followed by the formation of a specialized nitrogen-fixing organ called a symbiotic nodule [5,6]. The composition of the indigenous rhizobia population in the soil is quantitatively and qualitatively different, and pea roots are usually exposed to several compatible strains. At the same time, the choice of a microsymbiont may be determined not only by the presence of a particular bacterium but also by its competitiveness, and effective colonization of the roots is not necessarily associated with optimal nitrogen fixation [7,8].

There is an interest in using nitrogen-fixing symbiosis in agricultural practice to reduce the mineral load on the soil and accelerate the transition to adaptive agriculture. However, for the profitability of such an approach, symbiotic systems must consistently show high productivity, which cannot be achieved by relying solely on indigenous rhizobia in the soil. Biofertilizers based on specially selected highly effective strains can be a solution here, but under normal circumstances, such strains are likely to be suppressed by a more competitive native microbiota [9,10]. Thus, to give the selected strains a competitive advantage, a mechanism for the strict regulation of nodulation is needed. Such a mechanism was discovered more than 80 years ago in natural populations of pea from the Middle East.

In the 1930s of the last century, Soviet researcher Z.G. Razumovskaya, for the first time, demonstrated the inability of some pea samples from Afghanistan to form nodules in the soils of the Leningrad Oblast of Russia [11]. Subsequently, T.A. Lie, who began studying Afghanistan peas in search of natural variations of genes involved in symbiosis, discovered several genotypes exhibiting defects in nodule formation or nitrogen fixation when interacting with rhizobia strains common in Europe. At the same time, they developed normal symbiosis with strains isolated from the soils of Turkey, Israel, and a number of other Middle Eastern countries [12,13]. The genetic determinant responsible for the manifestation of such a trait was called *Sym2* [12]. Pea genotypes with higher selectiveness toward rhizobia have the *Sym2^A^* allele (from “Afghan” cultivars), for example, cv. ‘Afghanistan’ [14,15], while genotypes with an average specificity have the *Sym2^E^* allele (from “European” cultivars). To establish symbiosis with the *Sym2^A^* pea, the rhizobial strain should possess an additional gene called *nodX* [16,17]. This gene encodes the enzyme acetyltransferase, which performs specific acetylation of a symbiotic signaling molecule (Nod factor). The *Rhizobium leguminosarum* strain TOM isolated from the soils of Turkey is considered to be a model strain able to interact with *Sym2^A^* peas [17]. Recently, a promising candidate gene for *Sym2* was discovered and named *LykX* [18]. This gene encodes a receptor kinase with LysM domains and possesses the polymorphic variants in the first exon that match the specificity phenotype (i.e., cv. ‘Afghanistan’ and other highly specific genotypes carry a unique combination of SNVs compared with a large set of “European” pea genotypes with a low specificity of interaction with rhizobia) [18].

In 1996, A. Kozik mapped *Sym2* in the linkage group (LG) I of the pea genome [19]. As part of this work, a number of introgression lines were created, introducing the *Sym2^A^* allele from cv. ‘Afghanistan’ into the genome of the “European” cultivars ‘Rondo’ and NGB1238. According to genetic mapping data, line A33.18 has the smallest insertion of an alien genome among all the resulting lines [20]. The increased symbiotic selectiveness of A33.18 compared with the parental cultivar ‘Rondo’ has been confirmed in pot inoculation experiments, making it a good model to study the altered specificity of symbiosis. The aim of the present work was to characterize the A33.18 line phenotype- and genome-wise and to learn how it behaves in the field while interacting with the indigenous microbiota and the inoculum containing the *nodX^+^* strain TOM.

## 2. Results

### 2.1. Phenotypic Characterization of A33.18 Introgression Line

**Figure 1:**
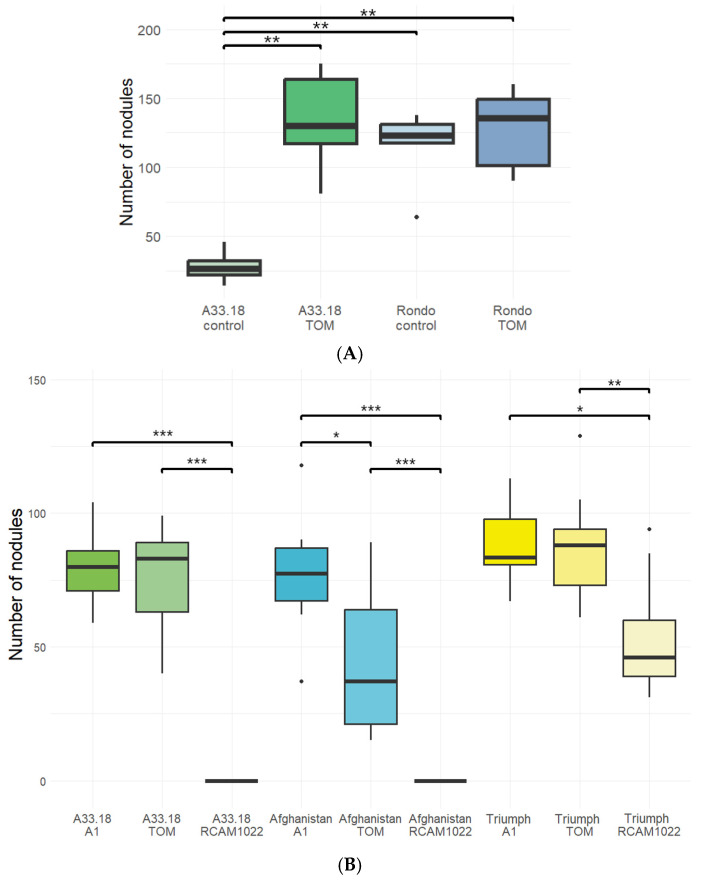
Nodulation phenotype of A33.18 line. (**A**)—nodulation in non-sterile soil (control and inoculation with TOM (*nodX^+^*) strain) (*n* = 6); (**B**)—nodulation in sterile sand (inoculation with RCAM 1022 (*nodX*^−^), TOM (*nodX^+^*), and A1 (*nodX^+^*) strains (*n* ≥ 5). *—*p*-value < 0.05, **—*p*-value < 0.01, ***—*p*-value < 0.001.

The introgression line A33.18 has the same habitus as its parental cultivar ‘Rondo’, i.e., it has shortened internodes (*le−*) and white flowers (*a*), as opposed to cv. ‘Afghanistan’, which has long internodes and purple flowers. With respect to the aboveground part, we failed to observe any phenotypic differences between A33.18 and cv. ‘Rondo’. Yet, A33.18 manifests the high specificity of interaction with rhizobia, which is inherent to cv. ‘Afghanistan’, as previously shown by A. Kozik [20]. We confirmed the high specificity phenotype of A33.18 in pot experiments with non-sterile soil (Ryazan Oblast, Russia) and sterile sand (Figure 1A,B and Figure S1, and Tables S1 and S2). A33.18 formed few nodules in non-sterile soil, while inoculation with *nodX^+^* strain TOM of *R. leguminosarum* restored the nodulation to the normal level; at the same time, cv. ‘Rondo’ developed a significant number of nodules both in the presence and absence of the strain TOM (Figure 1A). In sterile sand, when inoculated with the *R. ruizarguesonis* RCAM1022 strain (*nodX*^−^), A33.18 and cv. ‘Afghanistan’ did not develop any nodules, while cv. ‘Triumph’ (with the “European” type of symbiosis specificity [21]) demonstrated normal nodulation (Figure 1B). Inoculation with strains TOM or A1 (both *nodX^+^*) led to a significant number of nodules in all tested plant genotypes. Interestingly, inoculation with another *R. ruizarguesonis* strain, RCAM1026 (*nodX*^−^), in a separate experiment induced the formation of rare nodules on A33.18 roots, while cv. ‘Afghanistan’ still did not form any nodules (Figure S1; Table S3).

### 2.2. Characterization of Genome Composition of A33.18 Introgression Line

In order to estimate the portion of the genome that cv. A33.18 inherited from cv. ‘Afghanistan’, we sequenced the genomes of A33.18 and its parental cultivars ‘Rondo’ and ‘Afghanistan’ (Table S5) and then examined the polymorphic sites (single-nucleotide variants (SNVs) and insertions/deletions (indels)) by mapping the pre-processed reads onto the reference genome of the pea cv. ‘Frisson’ (NCBI accession number: JANEYU000000000). The genes containing these polymorphic sites were identified and subsequently localized to the genome.

The polymorphic sites common for A33.18 and cv. ‘Afghanistan’ but different in cv. ‘Rondo’ were included in further analysis. In total, we identified 244,235 such sites, including 236,007 SNVs and 8158 indels (Table S6). Since we were interested in estimating the number of variations affecting protein function, all variations associated with intergenic, intron up- and downstream regions were discarded from the further analysis. However, SNVs and indels in untranslated regions (UTRs) were kept as they may be associated with the regulatory regions (Table 1).

The newest assembly of the cv. ‘Frisson’ genome contained 47,753 annotated protein-coding genes. In total, 5001 variants (SNVs and indels) were found located in 464 protein-coding genes of A33.18 inherited from cv. ‘Afghanistan’. Furthermore, A33.18 inherited 14,365 genes from cv. ‘Rondo’, and other genes were non-polymorphic. Based on these figures, we estimated that A33.18 has inherited approximately 3.1% of its genome from cv. ‘Afghanistan’ (464/(464 + 14,365) × 100% = 3.1%).

**Figure 2:**
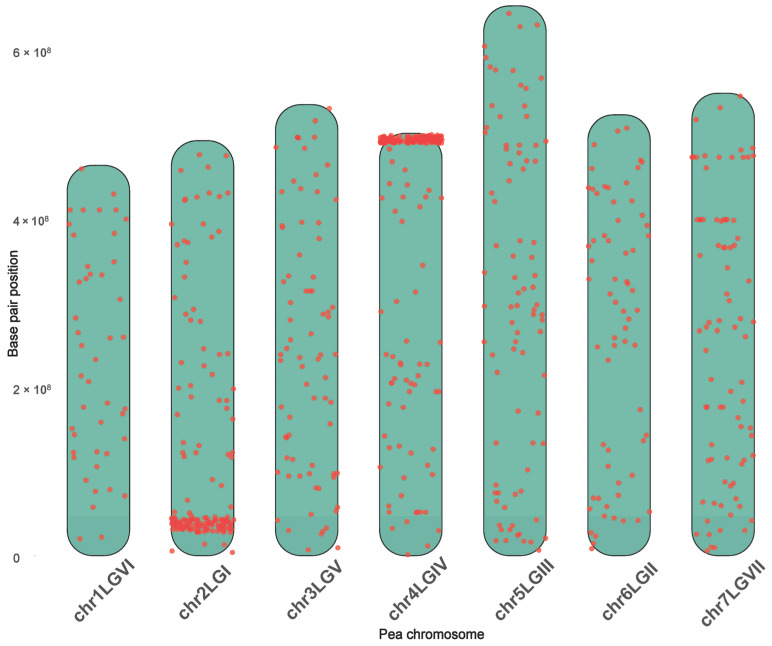
Distribution of SNVs and indels inherited by A33.18 from cv. ‘Afghanistan’ across the pea chromosomes.

The clearly visible region of the genome inherited by the A33.18 line from cv. ‘Afghanistan’ is located on chromosome 2 (linkage group I), which corresponds to the localization of the *Sym2* gene [19] (Figure 2). According to SNV/indel analysis, this region spans 18.2 Mb and contains 63 genes (Table S7). This region, indeed, contains the *Sym2* candidate gene *LykX* along with the paralogous receptor kinase genes *Sym37* and *K1*, all of which are involved in symbiosis with rhizobia [11]. In addition, we found two genes in this region with high similarity to the receptor kinase genes *LYK7* and *LYR4* of *Medicago truncatula* Gaertn., which currently are pending further analysis to draw any conclusions on their function in pea.

Interestingly, a second region inherited by A33.18 from cv. ‘Afghanistan’ was found to be located on the top of chromosome 4/LG IV (Figure 2), spanning 10.2 Mb and containing 75 genes. None of these genes encode receptor kinases, nor are they known to be involved in symbiosis with rhizobia. However, the putative function of many genes from this region is related to defense reactions/responses to pathogens (Table S7).

**Figure 3:**
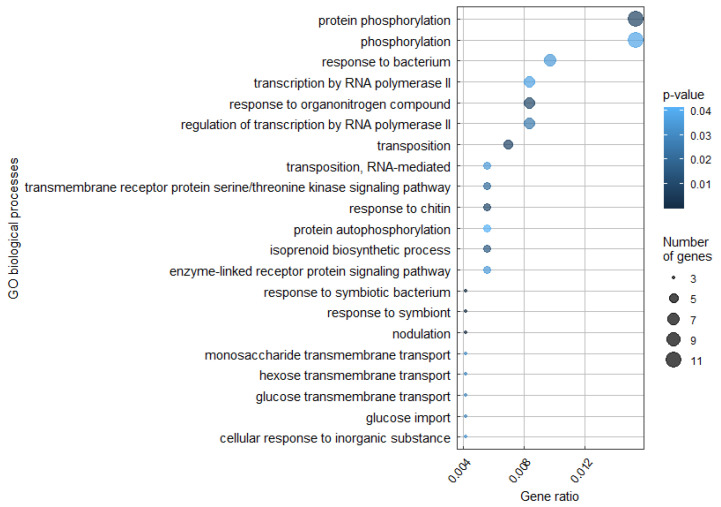
Gene enrichment analysis of all genes inherited by A33.18 from cv. ‘Afghanistan’. The point size is proportional to the number of genes in a particular group.

Functional analysis of the genes inherited by A33.18 from cv. ‘Afghanistan’ showed that those genes are associated with biological processes such as transcriptional regulation (i.e., transcriptional factors), protein phosphorylation, solute transport, and the response to pathogens. Moreover, most of the genes encode transferases, hydrolases, and oxidoreductases. Also, the inherited regions contain genes encoding proteins associated with nodulation (Figure 3). Some of these functional groups are characteristic of LysM kinase genes, so we can conclude that this result reflects the inheritance of the *Sym2* region, while the rest of the genes inherited from cv. ‘Afghanistan’ were distributed randomly across the genome.

In order to check whether other symbiotic receptor kinase genes from other parts of the genome could be inherited by A33.18 from cv. ‘Afghanistan’, we searched for the closest pea homologs of known LysM kinase genes from *M. truncatula* (listed in [22]). For 15 *Medicago* genes, we found the putative orthologs in the pea genome; for 3 genes, no sequences with high similarity were found in the pea genome of cv. ‘Frisson’; and for an additional 4 genes, the true pea orthologs could not be determined because they showed only short regions of similarity with pea genes of cv. ‘Frisson’. Among all the LysM receptor kinase genes, only *Sym37*, *K1*, *LykX*, and genes homologous to *LYK7* and *LYR4* of *M. truncatula* were inherited by A33.18 from cv. ‘Afghanistan’, while the others were inherited from cv. ‘Rondo’ (Table S8).

### 2.3. Manifestation of Specificity Trait of A33.18 Line in Field Conditions

#### 2.3.1. Development of Molecular Marker on Base of *nodX* Gene Sequence

The *Rhizobium leguminosarum* strain TOM has a unique sequence of the *nodX* gene, which differs in the protein-coding part by only two nucleotide changes from the most similar *nodX* sequence of the A1 strain (Figure S2). Other *nodX* sequences found by BLASTN search in the nucleotide and genome NCBI databases were either identical to that of the A1 strain or more distant from the *nodX* of TOM (Figure S2). The next most identical sequence belonged to the *Rhizobium bangladeshense* strain PLR8-1a plasmid p3 (CP071621.1) and had 24 nucleotide differences from the *nodX* of TOM, which resulted in 97.83% similarity.

**Figure 4:**
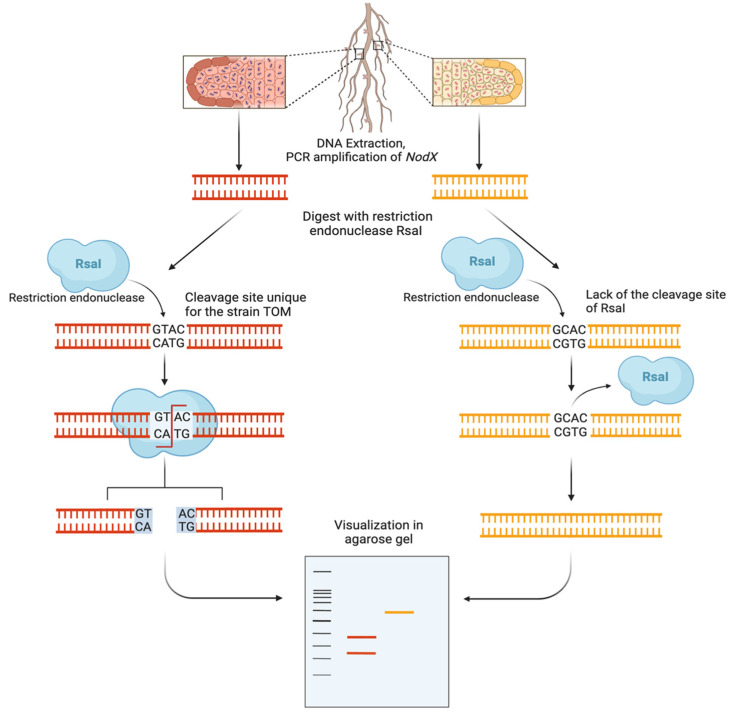
A scheme of the PCR and restriction-based analysis of the allelic state of the *nodX* gene.

Taking this into account, we developed a PCR-based marker distinguishing the alleles of the *nodX* gene after endonuclease RsaI digest. We designed the pair of primers specific to *nodX* of both TOM and A1 (but not to other rhizobial *nodX* sequences) and selected the endonuclease RsaI that recognizes the GATC site, which is present only in TOM’s but not in A1′s *nodX* gene sequence due to a single-nucleotide polymorphism (Figure 4). An example on agarose gel is given in Figure S3.

#### 2.3.2. Nodulation and Growth Parameters of A33.18 Line and cv. ‘Rondo’ in Field Experiment

**Figure 5:**
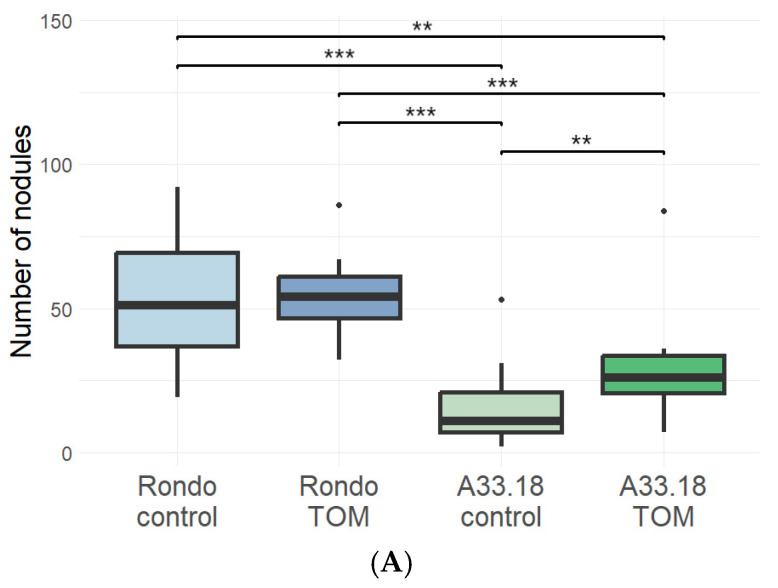

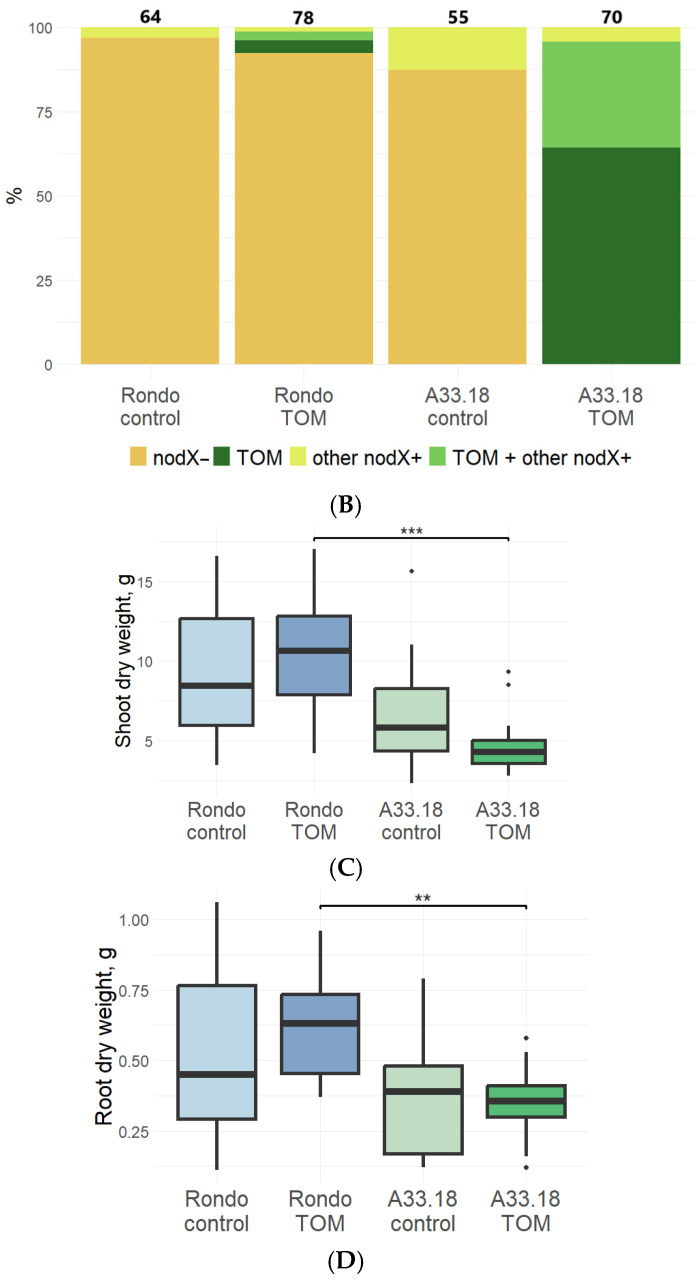
Nodulation and growth parameters of A33.18 and cv. ‘Rondo’ in the field experiment. (**A**)—nodulation (control and inoculation with TOM (*nodX^+^*) strain) (*n* ≥ 11), (**B**)—distribution of nodules formed by *nodX*^−^ and *nodX^+^* strains (the number of examined nodules is given above the columns), (**C**)—shoot dry weight (*n* ≥ 11), and (**D**)—root dry weight (*n* ≥ 11). **—*p*-value < 0.01, ***—*p*-value < 0.001.

The nodulation abilities of cv. ‘Rondo’ and the A33.18 line were tested in field conditions under inoculation with the *Rh. leguminosarum* TOM strain. Both in control conditions and under inoculation, A33.18 plants formed fewer nodules than the plants of cv. ‘Rondo’; inoculation with TOM increased the nodule number for A33.18 only (although cv. ‘Rondo’ formed significantly more nodules regardless of the inoculation) (Figure 5A; Table S4).

Analysis of the DNA extracted from individual nodules using the developed PCR-based marker allowed us to estimate the percentage of nodules formed by *nodX^+^* and *nodX*^−^ strains. In the control condition, 97% of nodules of cv. ‘Rondo’ were formed by *nodX*^−^ strains and 3% of nodules (2/64) by *nodX^+^* strains (having non-TOM alleles of *nodX*). Under inoculation with TOM, the nodules of cv. ‘Rondo’ still did not contain *nodX^+^* strains in 92% of cases, where 4% of nodules were identified as having a TOM *nodX* allele, 3% had a mixture of TOM and non-TOM *nodX* alleles, and 1% carried non-TOM *nodX* alleles, indicating the low competitiveness of TOM compared with indigenous rhizobia strains (Figure 5B).

The majority of A33.18 nodules in control conditions were also formed by *nodX*^−^ strains (87% by *nodX^−^* strains and only 13% by non-TOM *nodX^+^* strains), which indicates that the *nodX*-based specificity in A33.18 is not absolute (similar to the result described in Section 2.1). In turn, under inoculation with TOM, the fragment of the *nodX* gene was amplified from all but one of the A33.18 nodules. In 64% of cases, it belonged to the TOM strain; in 32%, there was a mixture of TOM and non-TOM *nodX^+^* strains; and 4% were identified as non-TOM *nodX^+^* strains (Figure 5B). Thus, the TOM strain successfully colonizes the nodules of the A33.18 line despite the presence of indigenous microflora in the soil.

Finally, the effect of inoculation with TOM on shoot and root weight at the stage of the analysis (7.5 weeks after planting/inoculation) was estimated. Although no significant difference was detected for shoots or roots of cv. ‘Rondo’ and A33.18 in control conditions, inoculation with TOM increased the plant weight for cv. ‘Rondo’ and decreased it for A33.18, so the difference between genotypes became statistically significant (Welch’s *t*-test: *p* < 0.001 for shoots and *p* < 0.01 for roots) (Figure 5C,D; Table S4). The observed trend for the decrease in the shoot and root dry weight of A33.18 due to inoculation with TOM, although not statistically significant, may suggest that nodules formed by TOM less effectively fix nitrogen than the nodules formed by indigenous strains. However, the reduction in the dry mass can also arise from many other unaccounted physiological, ecological, and environmental factors that directly or indirectly influence nodulation efficiency and nitrogen fixation.

## 3. Discussion

During evolution, legume plants acquired a complex genetic system controlling their interaction with rhizobia. An important role in such interaction is played by receptor kinase genes encoding receptors recognizing the structure of Nod factors. In the genome of *M. truncatula* cv. Jemalong A17, 22 such genes were described [22]. In pea cv. ‘Frisson’, their number is comparable. A natural polymorphism of one of these genes called *LykX* in pea is associated with the specificity of interactions with rhizobia, which makes it the prominent candidate gene for *Sym2* [18,23]. We showed here that introgression of the genomic region carrying *LykX* along with four related kinase genes by classical genetic methods (i.e., crossing and selection by phenotype) changes the specificity of interaction with rhizobia so that European pea plants become more specific, similar to the landraces from Afghanistan. Importantly, this specificity is not as strong in the introgression line A33.18 as in cv. ‘Afghanistan’, since A33.18 can form a few nodules with *nodX*^−^ strains. Perhaps other kinase genes that are different in ‘Afghanistan’ and ‘Rondo’, such as *Sym10* or the newly discovered homologs of *LYK7* and *LYR4*, may play a role in the manifestation of the specificity trait, but this issue needs further investigation.

In field conditions, A33.18, with an introduced 18.2 Mb region containing LysM kinase genes, interacted with the TOM strain more specifically than the parental cultivar ‘Rondo’. TOM was found in 7% of nodules of cv. ‘Rondo’ and in 96% of A33.18 nodules. At the same time, other *nodX*-containing strains were detected in A33.18 nodules after inoculation with TOM; 32% of nodules contained both TOM and non-TOM alleles of the *nodX* gene. This fact indicates that the *Sym2/nodX*-based system does not guarantee the exclusive penetration of the strain from biopreparation into the nodules of the *Sym2^A^* plants, as native *nodX*-containing strains are usually present in the soil. Probably, the genetic modification of the *Sym2* candidate gene sequence and simultaneous modification of the rhizobial genome (the introduction of the *nodZ* gene, which also helps overcome the *Sym2*-based specificity [24], instead of *nodX*) could enhance the specificity. Still, if the strain from biopreparation has a better nitrogen fixation ability than the indigenous strains, the appearance of other rhizobia in one-third of nodules may not be critical.

There is an idea that biopreparations should be made from local strains (as they are better adapted to local environmental conditions). Indeed, in white clover (*Trifolium repens* L.), only a small proportion of nodules was formed by the strain used in a biopreparation (from 8 to 18%, depending on the history of clover inoculation in the field), as opposed to inoculation with local strains, whereby this number increased up to 60% on average [25]. The *Sym2/nodX* system provides the opportunity to avoid this requirement since the introgression of the *Sym2^A^* allele into the plant’s genome deprives the plant of interactions with most of the native strains. Thus, even bad competitors (from the applied biopreparation) will penetrate the nodules. Indeed, under inoculation with TOM, less than 8% of nodules were formed by TOM on the roots of cv. ‘Rondo’ plants, but this number increased up to 95% when the plants (of the A33.18 line) were protected from the local strains by both *Sym2^A^* recessive alleles. This confirms previous data of Hogg and colleagues, according to which TOM, generally, is a poor competitor if not supported by the restrictive phenotype of *Sym2^A^* plants, mostly due to the smaller amount of produced Nod factor [8,26]. It should be noted that the use of the *Sym2/nodX* system in agriculture can lead to a local increase in the number of rhizobial strains containing the *nodX* gene, but this should not be of concern since *nodX* does not impair the interaction with plants lacking the *Sym2^A^* allele, and, given crop rotation, the increase will not be long-term. Meanwhile, a plant with the *Sym2^A^* allele will be at a disadvantage if there are not enough carriers of the *nodX* gene among the local rhizobia strains; however, the point of using such cultivars in agriculture is precisely to cut off all local microbiota (which may be suboptimal from the point of nitrogen fixation) and rely only on interactions with specially designed effective *nodX*-containing strains from the biopreparations.

In the field experiment, the number of nodules on the roots of A33.18 after inoculation with TOM at 7.5 weeks post-inoculation was lower than the number of nodules on the roots of cv. ‘Rondo’, which indicates that the rate of nodule formation by TOM may be lower than by other rhizobial strains. Another possible explanation for the low number of nodules on the roots of A33.18 may be due to the fact that receptor kinases perceive the Nod factor in the form of a heterodimer. One component of this heterodimeric complex in pea is Sym10, while another component may be either Sym37, K1, or LykX [11]. Since we showed that *Sym10* in A33.18 was inherited from cv. ‘Rondo’, while the second part of the heterodimeric receptor complex was inherited from cv. ‘Afghanistan’, this possible incompatibility of receptor parts may cause problems with Nod factor recognition that is expressed in a decrease in the nodule number and/or a delay in nodule development.

It is important to note that the traits of specificity and nitrogen fixation efficiency are often not linked in rhizobia [27], so the most specific strains may be poor nitrogen fixers and vice versa. This is apparently the case for TOM, which did not increase the weight of A33.18 plants when it inhabited the nodules (however, cv. ‘Rondo’ plants positively reacted to inoculation with TOM, which may point toward its possible plant growth-promoting activity or ability to ‘prime’ the plants for interaction with indigenous microflora). Therefore, in the next experiments, we plan to use other strains that are more efficient in N_2_ fixation and assess the influence of the indigenous microbiota on pea nodulation in the field using metagenomic approaches.

## 4. Materials and Methods

### 4.1. Plant Genotypes, Bacterial Strains, and Growth Conditions

The pea genotypes used in the study are listed in Table 2. The rhizobial strains are listed in Table 3.

Inoculation experiments were performed in a VB 1014 (Vötsch Industrietechnik, Balingen, Germany) growth chamber under the following climatic conditions: 16/8 h day/night, a temperature of 21 ± 1°C, a relative humidity of 75%, and an illumination of 600 μmol photons m^−2^ s^−1^. The pea seeds were surface-sterilized with concentrated sulfuric acid (10 min), washed 5–10 times with autoclaved tap water, and planted on Petri dishes on 1% agar for germination. After 3 days at room temperature in the dark, the germinated seeds were transferred into 2 L metal pots filled with a substrate. Depending on the experiment, the pots were either filled with quartz sand and heat-sterilized (200 °C) or filled with non-sterile soil (chernozem soil from a garden where peas were not cultivated for at least 15 years; Pronsk, Ryazan Region, Russia, Google map coordinates: 54.1031, 39.6041). Inoculation was performed during planting, with a culture of rhizobial strains grown on solid 79 medium for 3 days at 28 °C and resuspended in distilled water to a concentration of 107 colony-forming units (CFUs) per liter, with 150 mL of the rhizobium suspension per pot. In the experiments with quartz sand, a nitrogen-free mineral nutrition solution was added in an amount of 100 mL per 2 L pot (2 mM KH_2_PO_4_; 2 mM Ca_3_(PO_4_)_2_; 4 mM MgSO_4_; 5 mM K_2_SO_4_; 73.6 μM H_3_BO_3_; 18.3 μM MnSO_4_; 1.53 μM ZnSO_4_; 0.64 μM CuSO_4_; 0.2 μM (NH_4_)_2_MoO_4_; 0.21 μM CoCl_2_; and 37.9 μM NaFe-EDTA). The plants were watered with autoclaved tap water as needed.

The non-parametric Kruskal–Wallace test was used for the comparison of nodule counts.

### 4.2. Field Experiment

The field experiment was conducted during the summer of 2023 (from 15 May to 6 July) in the field of the N. I. Vavilov All-Russian Institute of Plant Genetic Resources (VIR) (St. Petersburg, Russia, Google map coordinates: 59.7099, 30.4289). The seeds of cv. ‘Rondo’ and A33.18 were planted in a completely randomized design, with three replicates per genotype per inoculation and 30 seeds per replicate. For inoculation, 100 seeds of a particular genotype were placed in a plastic bag and supplemented with 5 mL of either tap water or a water suspension of the *Rhizobium leguminosarum* TOM strain (10^8^ colony-forming units (CFUs) per liter). After 7.5 weeks post-planting and inoculation, at the onset of flowering, the plants were uprooted, dried at 60 °C, and weighed, while the fresh nodules were counted and collected for further analysis.

Statistical analysis was carried out by Welch’s *t*-test. The nodule count data were ln-transformed to conform to a normal distribution. All the visualization was performed using the ggplot2 package (ver. 3.5.1) in the R programming language (ver. 2024.12.0+467).

### 4.3. DNA Extraction from Nodules

The nodules from plants from one replicate were mixed, surface-sterilized with 96% ethanol, placed in cryotubes with glycerol, and stored at −80 °C. For DNA extraction, individual nodules were ground with a sterile spatula in 400 µL of LBX buffer in 1.5 mL Eppendorf tubes and heated at 65 °C for 20 min. Furthermore, 400 µL of chloroform was added to each sample, the samples were centrifuged for 5 min at 12,000× *g*, and the upper phase was transferred into a new tube. Then, isopropanol was added to the samples (0.9 volume), and the samples were mixed by inverting and centrifuged for 5 min at 12,000× *g*. The supernatants were discarded, and 400 µL of cold 70% ethanol was added to the samples. The samples were centrifuged for 5 min at 12,000× *g*, and the precipitate was air-dried and dissolved in Milli-Q water. The DNA extracts were stored at −80 °C.

### 4.4. PCR-Based Analysis of the Presence of the nodX Gene and Its Allelic State

Each DNA sample was used for the amplification of fragments of rhizobial genes *nodE* (the control, the gene present in all rhizobia) and *nodX* (the gene present in a small subset of rhizobial strains). The primers for the *nodX* gene matched the *nodX* sequence of the strains TOM and A1 and were not specific for *nodX* sequences of more distant rhizobial strains. The sequences of the primers are listed in Table 4. PCR was conducted in iCycler (Bio-Rad, Hercules, CA, USA) using ScreenMix-HS (Evrogen, Moscow, Russia) with the *nodE* and *nodX* primers separately. The proportions for the PCR reaction were as follows: ScreenMix-HS—2 μL; forward primer (3.3 μM)—0.75 μL; reverse primer (3.3 μM)—0.75 μL; DNA sample—1 μL; and H_2_O—5.5 μL. The conditions for the PCR reaction were as follows: 95 °C—5 min; 35 cycles of 95 °C—30 s, 64 °C—30 s, 72 °C—30 s; and 72 °C—5 min. The results of PCR were visualized on 1% agarose gel. The successful amplification of the *nodE* gene fragment and the absence of the *nodX* PCR product suggested that the particular nodule did not contain rhizobial strain(s) with *nodX.*

For the identification of the allelic state of the *nodX* gene, the PCR products were digested with RsaI endonuclease (Sibenzyme, Novosibirsk, Russia) and analyzed in 2% agarose gel. Due to the presence of the RsaI recognition site in the *nodX* sequence of the TOM strain, the fragment amplified on the DNA of TOM (and the DNA of nodules containing TOM) was cut into two fragments. The other known allele of the *nodX* gene inherent to the A1 strain (which is a local strain since it was isolated from the soil in St. Petersburg, formerly Leningrad [31]) did not contain such a restriction site; therefore, the amplicon remained undigested after the RsaI treatment.

### 4.5. Whole-Genome Sequencing and Reads Preprocessing

Shotgun genomic libraries were generated using the TruSeq DNA PCR-Free Kit from the DNA of A33.18, cv. ‘Afghanistan’ and cv. ‘Rondo’. The libraries were sequenced on the Illumina NovaSeq 6000 (Illumina, San Diego, CA, USA) platform at the Sirius University of Science and Technology, Russia. The paired-end reads generated were 150 base pairs in length. The raw-sequencing data have been deposited in the National Center for Biotechnology Information (NCBI) Sequence Read Archive (SRA) database under the accession number PRJNA1173591.

The quality of the raw-sequencing data was assessed using the FastQC software (https://www.bioinformatics.babraham.ac.uk/projects/fastqc/ (accessed on 15 October 2024)). Subsequently, the raw-sequencing reads were preprocessed to remove adapters and low-quality sequences using the BBDuk tool from the BBTools package (https://sourceforge.net/projects/bbmap/ (accessed on 15 October 2024)). For each genome, high-quality FastQ reads were mapped onto a reference genome of the cv. Frisson (NCBI accession number JANEYU000000000) using the bwa mem algorithm [32] with the default settings. On average, more than 90% of the reads were unambiguously mapped onto the reference.

### 4.6. SNV Calling and Identification of Genomic Regions Inherited by A33.18 from Afghanistan

SNVs and indels were called using bcftools [33] with the settings for diploid genomes. Low-quality variants were filtered out using a filter based on the bcftools command “view -i ‘%QUAL >= 20’”, followed by an analysis of the effects of variants on gene and protein structures using SnpEff [34]. For subsequent analysis, any variants found in intergenic, upstream, downstream, or intronic regions were excluded from the dataset.

To identify genomic regions that were inherited by cv. A33.18 from cv. ‘Afghanistan’, only allelic states that were common to both A33.18 and cv. ‘Afghanistan’ but different from those in cv. ‘Rondo’ were retained in the dataset. The chromosome associations of assembled contigs and scaffolds were determined based on their homology to the *P. sativum* ZW6 genome assembly [35].

### 4.7. Functional Analysis of Genes from Genomic Regions Inherited by A33.18 from cv. ‘Afghanistan’

The functional annotation of *P. sativum* genes was performed using Mercator4 [36] version 6.0 and EggNog [37]. GO enrichment analysis was conducted using the topGO R package (version 2.5.4) (https://bioconductor.org/packages/release/bioc/html/topGO.html (accessed on 15 October 2024)), using the weight01 algorithm and Fisher’s exact test. Visualization was accomplished using the ggplot2 R package (version 3.4.4) [38].

## 5. Conclusions

Here, we showed that the introgression of two recessive *Sym2^A^* alleles from wild Afghanistan pea into the genome of a European cultivar enabled the selective assembly of the super-organismal system (pea + rhizobia) in the field. The *nodX* gene that provides the specificity from the microorganism’s side is not frequent in native strains of *Rh. leguminosarum* or *Rh. ruizarguesonis* (common symbionts of pea) in Russia, which makes this system useful for Russian agriculture. However, the high specificity of interaction should be combined with the high nitrogen fixation activity of the rhizobial strain used as a biopreparation in order to guarantee high yields of peas. Accordingly, new strains combining high specificity with high nitrogen fixation are needed (the work on the selection and genetic modification of such strains is ongoing). Finally, it seems promising to increase the specificity of the interaction of pea with rhizobia via CRISPR/Cas9 editing of the *LykX* gene, for example, by the replacement of one or several nucleotides in the gene sequence encoding the receptor domain of the protein by the ‘prime-editing’ approach.

## Figures and Tables

**Table 1:** Locations of SNVs and indels that A33.18 inherited from cv. ‘Afghanistan’.

Region	Number of SNVs	Number of Indels
5′ UTR	164	23
Exon	4219	49
3′ UTR	504	42

**Table 2:** Pea genotypes used for genomic and phenotypic characterization of effect of *Sym2^A^* introgression from ‘Afghan’ to ‘European’ cultivars.

Line	Origin	Interaction with the *nodX*^−^ Strains	Interaction with the *nodX^+^* Strains	References
A33.18	The Netherlands	−/+	+	[20]
cv. ‘Rondo’	The Netherlands	+	+	[13]
cv. ‘Afghanistan’ (=NGB2150, JI1357)	Afghanistan	−	+	[14,15]
cv. ‘Triumph’	Russia	+	+	[21]

**Table 3:** Rhizobial strains used for inoculation of pea plants in pot and field experiments.

Strain	Species	Origin	Features	Genomic Sequence (NCBI Bioproject)	References
TOM	*Rhizobium leguminosarum* bv. *viciae*	Turkey	*nodX^+^*	PRJNA80887	[13,16,17]
A1	*Rhizobium leguminosarum* bv. *viciae*	Russia	*nodX^+^*	PRJNA609819	[28]
RCAM1022	*Rhizobium ruizarguesonis* (formerly *Rhizobium leguminosarum* bv. *viciae*)	Russia	*nodX* ^−^	PRJNA1038702	[29]
RCAM1026	*Rhizobium ruizarguesonis* (formerly *Rhizobium leguminosarum* bv. *viciae*)	Kazakhstan	*nodX* ^−^	PRJNA354725	[30]

**Table 4:** Primers used for amplification of *nodE* and *nodX* genes.

Gene	Sequence, 5′–3′
*nodE*	CAC CGA TGC TTC CTC TAT CTG GAC
	AAC GCA AGG ACA GCA TTC AT
*nodX*	GAT GAA TGC CAC TTT CAC AGT AAG
	CAG ATA CTG CAA GAT GCC GGT A

## Data Availability

The DNA-sequencing data have been uploaded to the NCBI under BioProject accession number PRJNA1173591.

## Supplementary Materials

The following supporting information can be downloaded at https://www.mdpi.com/article/10.3390/plants14030427/s1. Figure S1: Nodulation phenotype of A33.18 line compared with its parental cultivars ‘Afghanistan’ and ‘Rondo’; Figure S2: Alignment of *nodX* gene-coding sequences; Figure S3: Analysis of *nodX* gene allelic state; Table S1: Nodulation of ‘Rondo’ and A33.18 in non-sterile soil after inoculation with TOM strain; Table S2: Nodulation of A33.18, cv. ‘Afghanistan’ and cv. ‘Triumph’ in sterile sand after inoculation with *Rhizobium* sp. strains A1, TOM, and RCAM1022; Table S3: Nodulation of ‘Rondo’ and A33.18 in sterile sand after inoculation with *Rhizobium* sp. strains A1, TOM, and RCAM1022; Table S4: Growth and nodulation parameters of A33.18 and cv. ‘Rondo’ plants from field experiment; Table S5: Number of obtained sequencing reads; Table S6: Polymorphic sites common for A33.18 and cv. ‘Afghanistan’; Table S7: Genes inherited by A33.18 from cv. ‘Afghanistan’; Table S8: Pea genes encoding LysM kinases.
