# Supplementary material for: The Genomic and Phenotypic Characterization of the Sym2A Introgression Line A33.18 of Pea (Pisum sativum L.) with the Increased Specificity of Root Nodule Symbiosis
Source: Plants (Basel). 2025 Feb 1;14(3):427. doi: 10.3390/plants14030427 (PMC11821192; doi:10.3390/plants14030427)
Supplement: Supplementary file 1 [file plants-14-00427-s001.zip › Captions for Supplementary Figures and Tables_new.pdf]

Genomic and Phenotypic Characterization of the $Sym2^A$ Introgression Line A33.18 of Pea (*Pisum sativum* L.) with the Increased Specificity of Root Nodule Symbiosis

Anton S. Sulima, Igor Yu. Zhuravlev, Elizaveta A. Alexeeva, Marina S. Kliukova, Evgeny A. Zorin, Valeria A. Rakova, Michail L. Gordon, Olga A. Kulaeva, Daria A. Romanyuk, Gulnar A. Akhtemova, Aleksandr I. Zhernakov, Elena V. Semenova, Margarita A. Vishnyakova, Igor A. Tikhonovich and Vladimir A. Zhukov

Supplementary Figures and Tables.

Figure S1. Nodulation phenotype of A33.18 line as compared to its parental cultivars ‘Afghanistan’ and ‘Rondo’.

Figure S2. Alignment of the *nodX* gene coding sequences.

Figure S3. Analysis of the *nodX* gene allelic state.

Left lane in each row - DNA Ladder 100+ bp (Evrogen, Moscow, Russia).

Table S1. Nodulation of Rondo and A33.18 in non-sterile soil after inoculation with the TOM strain

Table S2. Nodulation of A33.18, cv. Afghanistan and cv. Triumph in sterile sand after inoculation with Rhizobium sp. strains A1, TOM and RCAM1022

Table S3. Nodulation of Rondo and A33.18 in sterile sand after inoculation with Rhizobium sp. strains A1, TOM and RCAM1022

Table S4. Growth and nodulation parameters of A33.18 and cv. Rondo plants from the field experiment

Table S5. Number of obtained sequencing reads.

Table S6. Polymorphic sites common for A33.18 and cv. ‘Afghanistan’.

Table S7. Genes inherited by A33.18 from cv. 'Afghanistan'.

Table S8. Pea genes encoding LysM kinases.
