# Supplementary figures and images for: The Genomic and Phenotypic Characterization of the Sym2A Introgression Line A33.18 of Pea (Pisum sativum L.) with the Increased Specificity of Root Nodule Symbiosis
Source: Plants (Basel). 2025 Feb 1;14(3):427. doi: 10.3390/plants14030427 (PMC11821192; doi:10.3390/plants14030427)

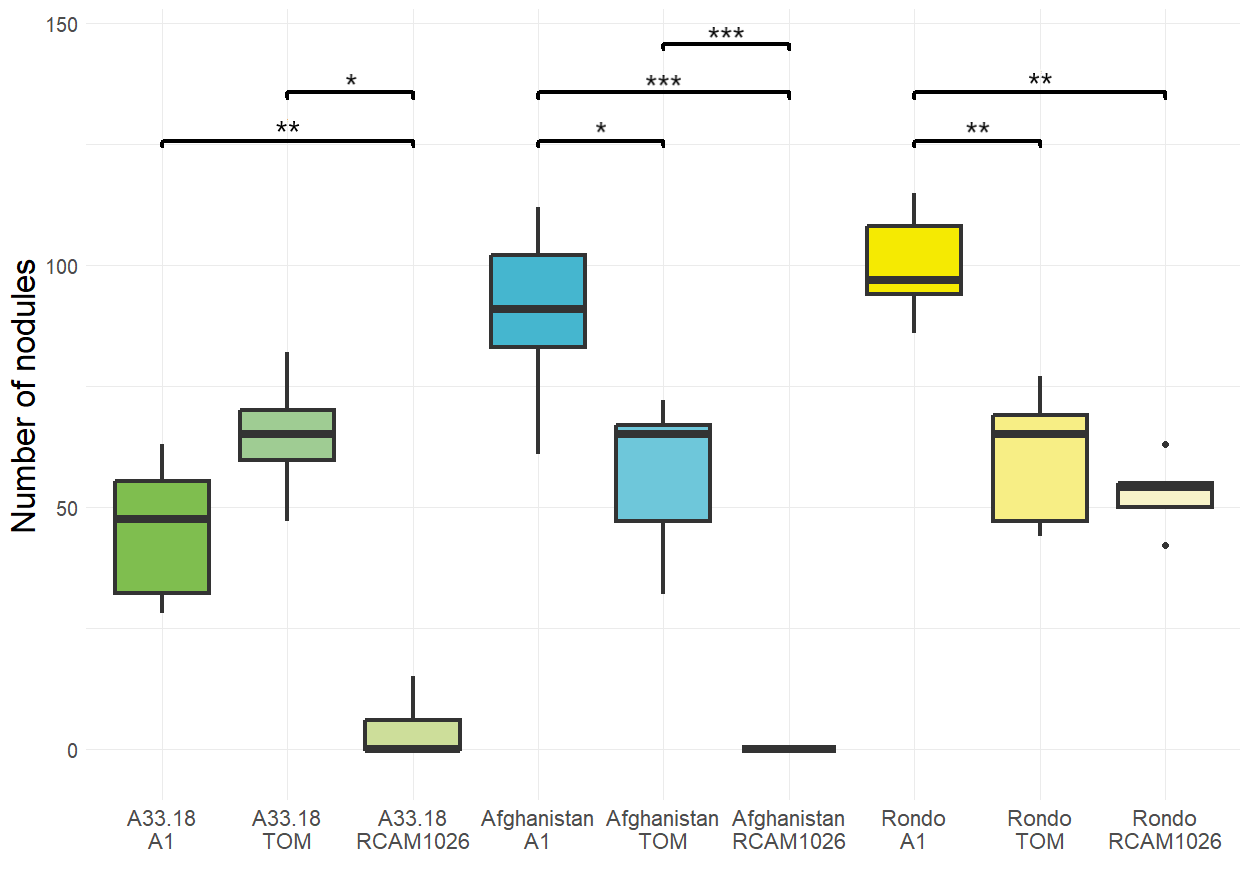

Supplement: Supplementary file 1 [file plants-14-00427-s001.zip › Figure S1.png]

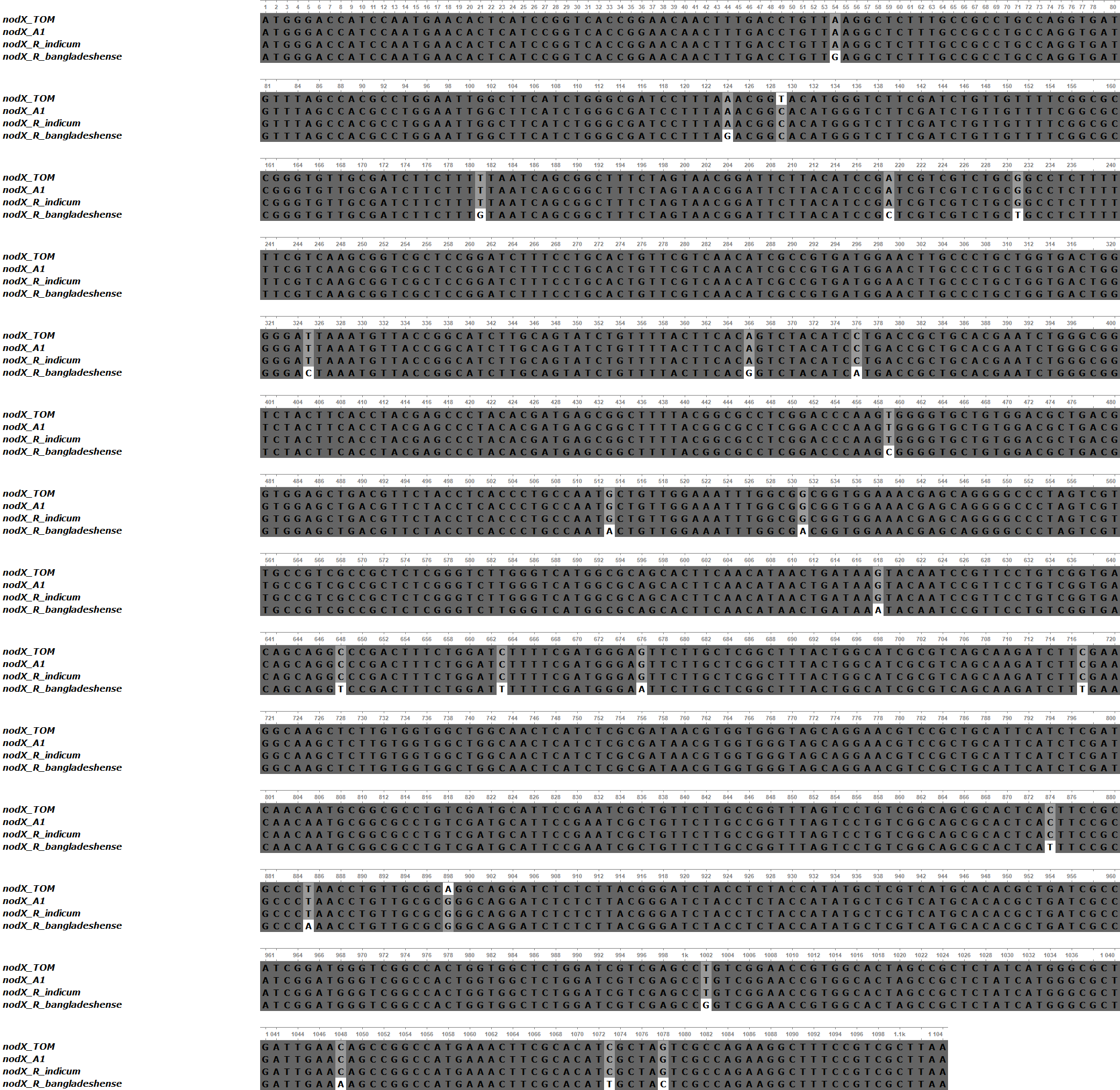

Supplement: Supplementary file 1 [file plants-14-00427-s001.zip › Figure_S2.png]

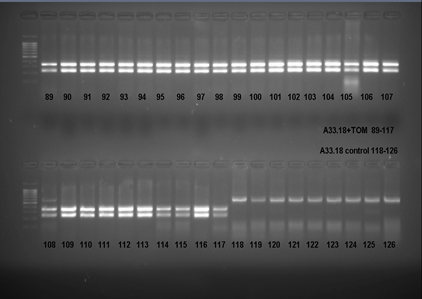

Supplement: Supplementary file 1 [file plants-14-00427-s001.zip › Figure_S3.png]
